# Global Optimization Employing Gaussian Process-Based Bayesian Surrogates†

**DOI:** 10.3390/e20030201

**Published:** 2018-03-16

**Authors:** Roland Preuss, Udo von Toussaint

**Affiliations:** Max-Planck-Institute for Plasma Physics, EURATOM Association, 85748 Garching, Germany

**Keywords:** parametric studies, global optimization, gaussian process, 02.50.-r, 52.65.-y

## Abstract

The simulation of complex physics models may lead to enormous computer running times. Since the simulations are expensive it is necessary to exploit the computational budget in the best possible manner. If for a few input parameter settings an output data set has been acquired, one could be interested in taking these data as a basis for finding an extremum and possibly an input parameter set for further computer simulations to determine it—a task which belongs to the realm of global optimization. Within the Bayesian framework we utilize Gaussian processes for the creation of a surrogate model function adjusted self-consistently via hyperparameters to represent the data. Although the probability distribution of the hyperparameters may be widely spread over phase space, we make the assumption that only the use of their expectation values is sufficient. While this shortcut facilitates a quickly accessible surrogate, it is somewhat justified by the fact that we are not interested in a full representation of the model by the surrogate but to reveal its maximum. To accomplish this the surrogate is fed to a utility function whose extremum determines the new parameter set for the next data point to obtain. Moreover, we propose to alternate between two utility functions—expected improvement and maximum variance—in order to avoid the drawbacks of each. Subsequent data points are drawn from the model function until the procedure either remains in the points found or the surrogate model does not change with the iteration. The procedure is applied to mock data in one and two dimensions in order to demonstrate proof of principle of the proposed approach.

## 1. Introduction

The complex physics model in our case is given by the modelling of particle transport and plasma-wall interaction in the scrape-off layer of fusion plasmas, which is carried out numerically by the interplay of two extensive codes either describing the plasma solving a fluid equation or the transport of neutrals by a Monte-Carlo method. Each code part produces data sets that the other part of the code needs to process—a circumstance which leads to running times in the order of weeks. Still, after years of computer runs for multiple parameter settings, quite a large database has been gathered with over 1500 entries. With these data at one’s disposal one is tempted to employ some surrogate modelling in order to explore the data set for extrema in certain data ranges motivated by physics, or simply to give advice for which parameter setting the next computer run has to be executed in order to increase the information content of the database most effectively.

A long established method in global optimization of complex multi-modal models is the construction of a response surface via fast surrogate models [1]. The numerically easy accessible surrogate is employed to find the maximum of the response surface whose coordinates are fed back to the original function. With the outcome obtained the surrogate model gets re-parameterized and the whole procedure is iterated until success. Unfortunately, a lot of pitfalls are out to spoil the result by pretending delusive maxima of the surrogate model unrelated to the real maxima of the complex model [2]. We propose utilizing the prediction of function values by the Gaussian process method for the surrogate model and to profit from the capabilities of a Bayesian approach to self-consistently adjust hyperparameters according to the information present in the data. Moreover, we employ two different utility functions—expected improvement and maximum variance—to find the next parameter set at which the expensive model is asked for a new data point. The interplay of these algorithmic steps turns out to let the surrogate model successfully describe the optimum region of the unknown model behind the data as closely as possible.

The paper is organized as follows. Section 2, Section 3, Section 4 and Section 5 treat the analytics behind each algorithmic step mentioned above. Section 6 gives a description of the proposed algorithm and Section 7 demonstrates proof of principle in one and two dimensions. Summary and outlook complete the paper, while a notation table can be found in the appendix.

## 2. The Gaussian Process Method

The Gaussian process method has been appreciated much in the fields of neural networks and machine learning [3,4,5,6,7]. Building on this, further work showed the applicability of active data selection via variance based criteria [8,9]. In general, for unknown functions that are costly to evaluate, Bayesian optimization [10] was deployed either with sequential [1,11] or batch design [12], and recently as a combination of both [13,14]. The very first efforts in geosciences [15] tackling the problem above with so-called kriging [16] can be classed among the realm of Gaussian process methods as well. Advancement of the method exploited additional information on the variables used—so-called cokriging—and led to the construction of covariance matrices for multi-output regression based on the convolution of an input Gaussian process and a smoothing kernel [17,18]. This was generalized later on by Alvarez et al. [19,20] by designing smoothing kernels derived from physical principles. The presentation of the Gaussian process method in this paper was already introduced in [21], and follows in notation—and apart from small amendments—the very instructive book of Rasmussen & Williams [22].

The problem of predicting function values in a multi-dimensional space supported by given data is a regression problem for a non-trivial function of unknown shape. Given *n* input data vectors $x_{i}$ of dimension $N_{\dim}$ (with matrix $X=(x_{1},x_{2},\ldots ,x_{n})$) and corresponding target data $y={\left(y_{1},\ldots ,y_{n}\right)}^{T}$, blurred by Gaussian noise of variance $\sigma_{d}^{2}$, the sought quantity is the target value $f_{*}$ at test input vector $x_{*}$. The latter would be generated by a function f(x)

(1) y=f(x)+ϵ,

where 〈ϵ〉=0 and $\langle \epsilon^{2}\rangle =\sigma_{d}^{2}$. Since we are completely ignorant about the (complex) model describing function, our approach is to employ the Gaussian process method, with which any uniformly continuous function may be represented. As a statistical process it is fully defined by its covariance function and called Gaussian, because any collection of random variables produced by this process has a Gaussian distribution.

The Gaussian process method defines a distribution over functions. One can think of the analysis as taking place in a space of functions (function-space view) which is conceptually different from the familiar view of solving the regression problem of, for instance, the standard linear model (SLM)

(2) $$f^{\mathrm{SLM}}\left(x\right)=x^{T}w,$$

in the space of the weights w (weight-space view). At this point it is instructive to restate the results for the latter: the predictive distribution depending on mean ${\bar{f}}_{*}$ and variance for a test input data point $x_{*}$ is given by

(3) $$p\left(f_{*}^{\mathrm{SLM}}|X,y,x_{*}\right)\propto N\left({\bar{f}}_{*}^{\mathrm{SLM}},\mathrm{var}\left(f_{*}^{\mathrm{SLM}}\right)\right),$$

with

(4) $$\begin{array}{c} {\bar{f}}_{*}^{\mathrm{SLM}}=\frac{1}{\sigma_{d}^{2}}x_{*}^{T}{\left[\sigma_{d}^{-2}XX^{T}+\Sigma_{p}^{-1}\right]}^{-1}Xy, \end{array}$$

(5) $$\begin{array}{c} \mathrm{var}\left(f_{*}^{\mathrm{SLM}}\right)=x_{*}^{T}{\left[\sigma_{d}^{-2}XX^{T}+\Sigma_{p}^{-1}\right]}^{-1}x_{*}. \end{array}$$

$\Sigma_{p}$ is the covariance in a Gaussian prior on the weights. Now we transform these results to the function-space view of the Gaussian process method.

## 3. Prediction of Function Values

As stated above the defining quantity of the Gaussian process method is the covariance function. Its choice is decisive for the inference we want to apply. It is the place where we incorporate all the properties which we would like the (hidden) function describing our problem to have in order to influence the result. For example, the neighbourhood of two input data vectors $x_{p}$ and $x_{q}$ should be of relevance for the smoothness of the result. This should be expressed by a length scale λ which represents the long range dependence of the two vectors, where larger values correspond to a surrogate function as a best-fit curve, while for smaller values the surrogate tries to match each data point.

For the covariance function itself we employ a Gaussian type exponent with the negative squared value of the distance between two vectors $x_{p}$ and $x_{q}$

(6) $$k\left(x_{p},x_{q}\right)=\sigma_{f}^{2}\exp\left\{-\frac{1}{2}\frac{{\left(x_{p}-x_{q}\right)}^{T}\left(x_{p}-x_{q}\right)}{\lambda^{2}}\right\}.$$

$\sigma_{f}^{2}$ is the signal variance. If one is ignorant about this value, the literature proposes to set it to one as a default value (Chapter 2.3 and 5.4 in [22]). However, in probability theory, we consider it as a hyperparameter (see next chapter). To avoid lengthy formulae, we abbreviate the covariance matrix of the input data as ${\left(K\right)}_{ij}=k\left(x_{i},x_{j}\right)$ and the vector of covariances between test point and input data as ${\left(k_{*}\right)}_{i}=k\left(x_{*},x_{i}\right)$.

Moreover, we consider the degree of information which the data contain by a term $\sigma_{n}^{2}\Delta$ to be composed of an overall variance $\sigma_{n}^{2}$ accounting for data noise and the matrix Δ with the variances $\sigma_{d}^{2}$ of the given input data on its diagonal and zero otherwise. $\sigma_{n}^{2}$ is an overall factor that is supposed to catch global uncertainty and may be considered as a hyperparameter. On the other hand, the matrix entry ${\left(\sigma_{d}\right)}_{i}$ is the relative uncertainty estimation of a single data point $y_{i}$ and provided by the experimentalist. If no uncertainties of the input data are given, Δ is set to the identity matrix. It can be shown (Chapter 2.2 in [22]) that in analogy to Equation (3) for given λ, $\sigma_{f}$ and $\sigma_{n}$ the probability distribution for a single function value $f_{*}$ at test input $x_{*}$ is

(7) $$p\left(f_{*}|X,y,x_{*}\right)\propto N\left({\bar{f}}_{*},\mathrm{var}\left(f_{*}\right)\right),$$

with mean

(8) $${\bar{f}}_{*}=k_{*}^{T}{\left(K+\sigma_{n}^{2}\Delta \right)}^{-1}y,$$

and variance

(9) $$\mathrm{var}\left(f_{*}\right)=k\left(x_{*},x_{*}\right)-k_{*}^{T}{\left(K+\sigma_{n}^{2}\Delta \right)}^{-1}k_{*}.$$

## 4. Evaluation of the Hyperparameters

The hyperparameters $\theta ={\left(\lambda ,\sigma_{f},\sigma_{n}\right)}^{T}$ determine the result of the Gaussian process method. Depending on the data, their probability distribution in phase space could be multi-modal and spread over various regimes [23]. A comprehensive representation of the Gaussian process describing the data is therefore only achievable by taking into account the full phase space of the hyperparameters and marginalize over it [24]. However, searching for the maximum in the surrogate model with complete exploration of the phase space may turn out to be futile when it comes to real-time applications. Fortunately, a full description of the data by the Gaussian process method is not what we are looking for. We only want to find the global maximum of the data or of a function of them. Therefore, it will be sufficient to have a helpful value of each hyperparameter with which it is possible to get an adequate description in the region of the maximum of the surrogate. This is accomplished by the expectation value given by the first moment *m* = 1 of

(10) $$\langle \theta^{m}\rangle =\frac{1}{Z}\int d\theta \;\theta^{m}p\left(\theta |y\right)=\frac{1}{Z^{\prime }}\int d\theta \;\theta^{m}p\left(y|\theta \right)p\left(\theta \right)\;.$$

The result for the respective hyperparameter from Equation (10) is inserted to into Equations (8) and (9) to specify the surrogate. The second central moment $\langle \theta^{2}\rangle -{\langle \theta \rangle }^{2}$ represents the variance and is a diagnostic measure of the validity of the procedure by using only the expectation value instead of the full probability distribution of the hyperparameters.

Gaussian priors are employed for the hyperparameters with mean and variance one but constrained to be positive,

(11) $$p\left(\theta_{i}\right)\propto N\left(1,1\right)\;\;\forall \;\;\theta_{i}\geq 0\;\mathrm{and}\;p\left(\theta_{i}\right)=0\;\;\mathrm{otherwise}.$$

The marginal likelihood p(y|θ) is obtained by

(12) p(y|θ)=∫dfp(y|f,θ)p(f|θ).

As we deal with the Gaussian process the probability functions are of Gaussian type, with the likelihood as $p\left(y|f,\theta \right)\propto N\left(f,\sigma_{n}^{2}\Delta \right)$ and the prior for f as p(f|θ)∝N(0,K) [22]. Thus, the integration in Equation (12) yields

(13) $$\log p\left(y|\theta \right)=\mathrm{const}-\frac{1}{2}y^{T}{\left[K(\theta )+\sigma_{n}^{2}\Delta \right]}^{-1}y-\frac{1}{2}\log\left|K(\theta )+\sigma_{n}^{2}\Delta \right|\;.$$

Equations (11) and (13) constitute the sampling density for a Markov chain Monte Carlo (MCMC) calculation of Equation (10). The MCMC calculations were performed with binning to 10 groups, i.e., bins, of 200 evaluations each, separated again by 10 bins of 200 evaluations, which start from randomly chosen initial values (see, e.g., [25]).

## 5. Utility Functions

In a region of interest (RoI) set by the experimentalist we alternate among two well established, but conceptually different utility functions—expected improvement and maximum variance—and search the chosen one for its maximum. Each utility function gets input from the surrogate model represented by target value Equation (8) and its variance Equation (9) for a certain set of hyperparameters 〈θ〉 determined by Equation (10). Maximization of each respective utility function then returns a vector $\xi_{\max}\in$ RoI establishing a new parameter set at which a new expensive simulation is started. After that, with the new data point getting added to the existing data pool, we are out for the next proposal of a most promising parameter set and change to the other utility function to search for its optimum.

For a first utility function $U_{\mathrm{EI}}\left(\xi \right)$, we employ the expected improvement approach [2,26] which states how much improvement *I* of the maximum $f_{\max}$ of the surrogate model is going to be expected according to the probability distribution of the function values Equation (7),

(14) $$U_{\mathrm{EI}}\left(\xi \right)=\langle I\rangle =\int_{f_{\max}}^{\infty }f_{*}p\left(f_{*}|X,y,\xi \right)df_{*}\;.$$

Inserting Equation (7) into (14) and solving the integral gives readily

(15) $$U_{\mathrm{EI}}\left(\xi \right)=\sqrt{\frac{\mathrm{var}(f_{*})}{2\pi }}\exp\left(-u^{2}\right)+\frac{{\bar{f}}_{*}}{2}\left[1+\mathrm{erf}(u)\right]\;,\;\mathrm{with}\;u=\frac{{\bar{f}}_{*}-f_{\max}}{\sqrt{2\mathrm{var}(f_{*})}}\;.$$

**Figure 1:**
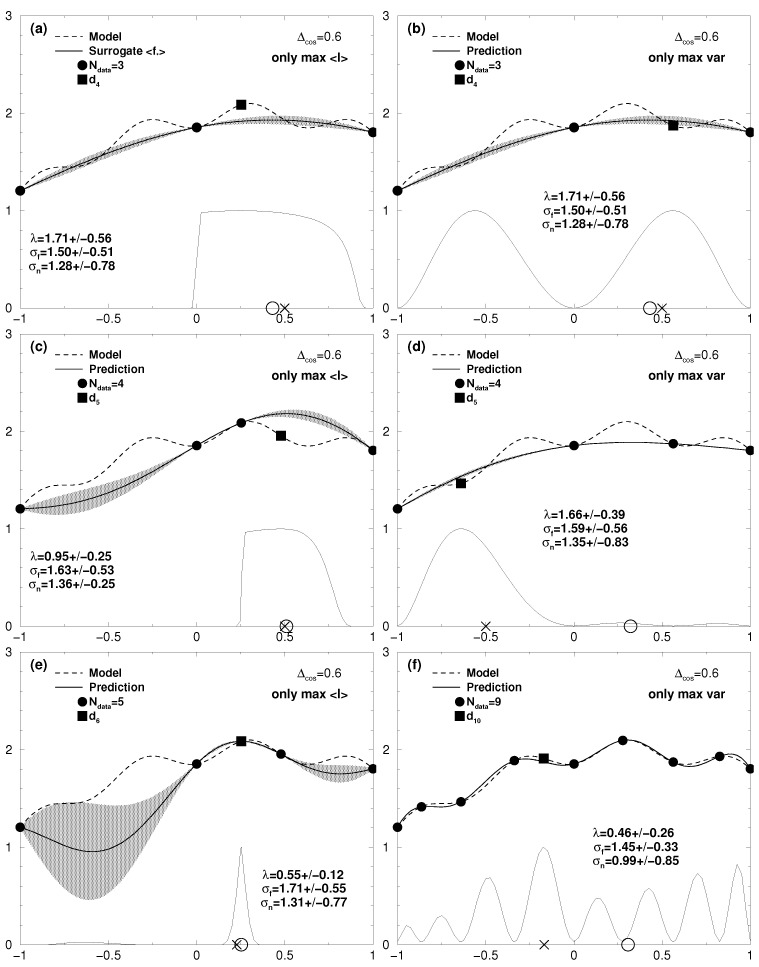
One-dimensional case: cosine fine structure with $\Delta_{\cos}$ = 0.6. On top of a broad parabola around *x* = 0.3 (dashed line, see Equation (18)). A standard error of $\sigma_{d}$ = 0.001 was assigned to the data. Left column: surrogate response (thick line) applying the algorithm with expected improvement utility $U_{\mathrm{EI}}$ only, (**a**) *N* = 3; (**c**) *N* = 4; (**e**) *N* = 5. Though in (e) the global optimum at *x* = 0.3 seems to be revealed, the surrogate still deviates with its maximum at *x* = 0.25. Further iterations (not shown) are kept locked in the points (and surrogate) shown. Right column: surrogate response (thick line) applying the algorithm with variance utility $U_{\mathrm{MV}}$ only, (**b**) *N* = 3; (**d**) *N* = 4; (**f**) *N* = 9. Though the variance (grey shadow) is misleadingly small, the algorithm may proceed in always finding a largest variance to propose a calculation of a data point with the true model. This finally succeeds to have the surrogate resembling the correct functional behaviour of the true model. The thin line at the bottom of each figure shows the respective utility scaled to [0:1]. The “X” on the base line represents the initial value proposed to the Powell search for the maximum of the respective utility function. At the obtained maximum the complex model is asked for an additional data point (filled square). The circle on the base line represents the highest value of the surrogate model revealed so far. The expectation values and standard deviations of the hyperparameters are stated in each figure.

**Figure 2:**
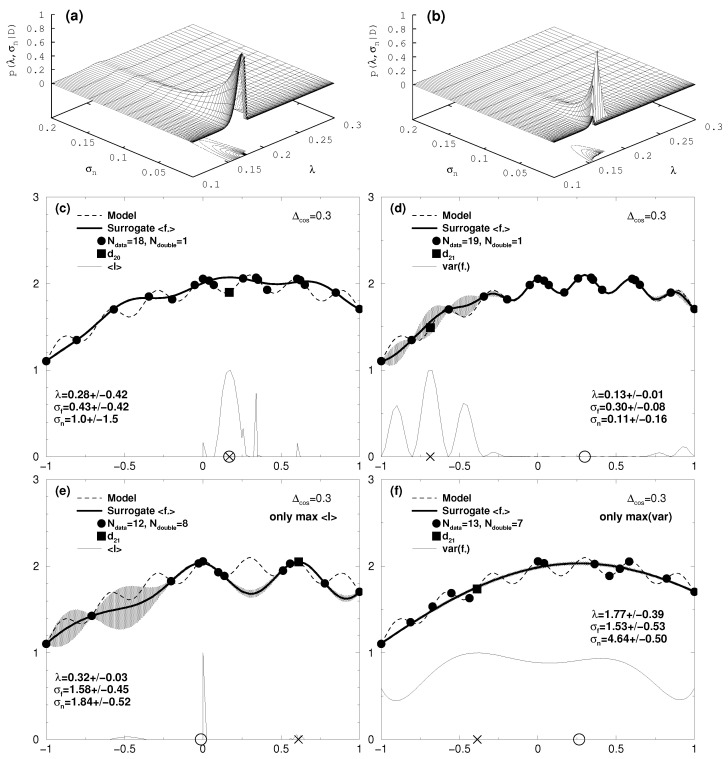
One-dimensional case: cosine fine structure with $\Delta_{\cos}$ = 0.3 on top of a broad parabola around *x* = 0.3 (dashed line, see Equation (18)). A standard error of $\sigma_{d}$ = 0.01 was assigned to the data. Top row: probability distribution for the hyperparameters λ and $\sigma_{n}$ for (**a**) *N* = 19 and (**b**) *N* = 20, corresponding to the middle row with the surrogate response in in (**c**,**d**) for iteration 19 and 20, respectively. The grey shaded area is the uncertainty range of the prediction, i.e., surrogate model (tick line), obtained as a Gaussian process with $N_{\mathrm{data}}$ data (filled circles). The thin line in the bottom of (**c**–**f**) shows the the utility functions $U_{\mathrm{EI}}$ in (**c**,**e**) and $U_{\mathrm{MV}}$ in (**d**,**f**). The “X” on the base line represents the initial value proposed to the Powell search for the maximum of the respective utility function. At the obtained maximum the complex model is asked for an additional data point (filled square). The circle on the base line represents the highest value of the surrogate model revealed so far. The expectation values and standard deviations of the hyperparameters are stated in each figure.

Optimizing this utility to find the global optimum of the true model via its surrogate has the known drawback [2] that the potential for deception by wrong estimation of the variance $\mathrm{var}(f_{*})$ is high. This leads to a perpetual choice of initial values close to existing data points and a change only after exhaustive calculations in the vicinity of them. New data points are acquired close to previous ones in the left or the right maximum, while the actual global maximum lies just in between. Apart from such deadlocks, the concept of looking at the expected improvement may work out superficially fine as seen in the left panel of Figure 1. Within a few iterations the global optimum of the true model function seems to be identified, while less important regions are neglected. However, the maximum shown by the surrogate at *x* = 0.25 is not at the position of the global optimum of the true model at *x* = 0.3. Moreover, most disappointingly, even with more and more iterations, the proposed positions to calculate a new data point are kept locked in the existing ones. An example of this behaviour may be found in Figure 2e where the surrogate for a Rastrigin-like function [27] is erroneously kept locked at two lower local maxima.

The other utility function we use is just the variance of the surrogate from Equation (9),

(16) $$U_{\mathrm{MV}}\left(\xi \right)=\mathrm{var}\left(f_{*}\right)\;.$$

Firstly, looking for the maximum of Equation (16) has nothing to do with looking for the global maximum of the surrogate model or of the true model. Improving the variance of the surrogate simply improves the description of the true model by the surrogate. However, with this iteratively improving surrogate model it is possible to reveal undiscovered regions in the true model otherwise kept hidden using other utilities. The procedure is shown top to bottom in the right panel of Figure 1. In searching for the maximum of the variance in the surrogate and getting a new data value from the true model, the surrogate resembles the true model in the end. Of course this takes way more iterative steps than, e.g., pursuing the expected improvement; however, it will help to avoid deadlocks.

To search the utility function $U_{\mathrm{EI}}$ or $U_{\mathrm{MV}}$ for its respective maximum at $\xi_{\max}$,

(17) $$\xi_{\max}=\arg\max_{\left\{\xi \right\}}U_{\mathrm{EI}/\mathrm{MV}}\left(\xi \right)\;,$$

we simply employ Powell’s method (see chapter 10.5 of [28]). We choose this method because it is easy deployed and, with a given initial value, very fast in finding the next local maximum. However, since Powell’s method does not guarantee to reveal the global maximum it is crucial to implement an iterative algorithm which sophisticatedly spreads the initial values to find not only the maxima of the respective utilities but also—in the end—the global optimum of the expensive model function.

## 6. Global Optimization Algorithm

The task of the algorithm is to get a surrogate model to represent a complex model function costly to evaluate with respect to certain input variables. A side condition is that the surrogate should provide the best possible representation of the model at least at the most important point we are looking for, i.e., the global optimum, and is allowed to be vague otherwise. Moreover, the algorithm should benefit from a combination of alternating utility functions and a sophisticated choice of initial values for the maximum search of the utilities. The dimension of the problem is set by the number of input variables.

In a real-world problem we would start with a set of data points already obtained from the complex simulations within a region of interest, which conveniently encloses the maximum we are looking for (surrogate modelling fails outside of the region supported by the data). Here, for demonstration purposes, we work with mock data drawn from a multi-dimensional Sobol sequence. Although real data may be spread less evenly over input space a Sobol sequence seems to describe best the attempts of an experimentalist to provide full coverage of parameter space. To ensure that the Gaussian process is unhampered by large scales and to that the data subjected to the numerical treatment is free of any bias, the input data is scaled to [−1:1] in all dimensions and the target data is whitened, i.e., scaling to [−1:1] and removal of a possible linear trend. Next the Gaussian process method is applied to determine a surrogate model given by Equation (8) and its variance Equation (9). It is specified by the expectation values of the hyperparameters 〈θ〉 to be calculated from Equation (10) by MCMC. To propose a new parameter set for the expensive model to result in a further data point, we alternate the maximum search among the utilities Equations (15) and (16). The surrogate is “back-whitened” to be fed into the utilities, which means in this case basically restoring the removed linear trend (if present). The routine used for finding the maximum employs just (inverse) line minimization in multiple dimensions, as performed by Powell’s routine [28]. However, the initial point has to be chosen carefully. For this we choose the maximal value of the respective utility function at all points being in the middle between all possible pairs of points in the data set and additionally at all data points themselves. Due to the properties of the Gaussian process, these are the locations where either the variances will be largest or the expected improvement is going to be most promising. Eventually, the optimization routine returns the position of a maximum found on the response surface. If this position lies within a certain range (we choose 0.5% of the RoI) of an already existing data point, we do not invoke the complex model but diminish the standard error of the existing target data value by a factor of $\sqrt{2}$. Otherwise, an additional data point is simulated with the complex model. The whole procedure is iterated until some stopping criterion is fulfilled, e.g., a newly found maximum differs from the previous one only within computationally accuracy.

## 7. Results in One and Two Dimensions

In order to demonstrate our algorithm we examine a Rastrigin-like function [27] with a broad maximum and a cosine structure with period $\Delta_{\cos}$ on top of it:

(18) $$y=2-\sum_{i=1}^{N_{\dim}}\left\{\frac{1}{2}{\left(x_{i}-0.3\right)}^{2}-\frac{1}{10}\cos\left[\frac{2\pi (x_{i}-0.3)}{\Delta_{\cos}}\right]\right\}\;.$$

The global maximum is set at 0.3, while the variability of the function within the RoI [−1:1] is given by the factor $\Delta_{\cos}$ which will be chosen in between 0.1 and 1.

### 7.1. One-Dimensional Results

Figure 2 shows a one-dimensional test case for $\Delta_{\cos}$ = 0.3.

Though this still seems to be a moderate variability with respect to the RoI, the model shows a lot of local extrema which constitute pitfalls for the search of the global maximum. From the initial data set of *N* = 3 already the data point for *x* = 0 represents a local maximum which is hard to overtop for methods ignoring the uncertainty of the surrogate model (see [2]). And indeed, as can be seen in Figure 2e which we already discussed in Section 5, just using the expected improvement Equation (15) as the utility function would not reveal the global optimum at *x* = 0.3, even if the number of iterations is driven far beyond the displayed case for 17 additionally acquired data. The graphs Figure 2c,d show iteration *N* = 19 and *N* = 20 with the proposed algorithm. The iteration *N* = 19 was performed with the expected improvement utility $U_{\mathrm{EI}}$, while at iteration *N* = 20 we switched to the variance utility $U_{\mathrm{MV}}$. With the data point $d_{20}$ acquired in Figure 2c the resulting Gaussian process reveals in Figure 2d the sought for cosine fine structure around the global optimum at *x* = 0.3. This is not only accompanied by a reduction of the expectation value of the hyperparameter λ from 0.26 to 0.13, but also by a remarkable change in the shape of the hyperparameter distribution, as shown in the top row of Figure 2. Indeed, the mode in the distribution for λ becomes particularly pronounced, reflected by a decreased standard deviation from 0.42 to 0.01. Here the algorithm benefits from the self-adjusting skills of the Bayesian approach to adjust the hyperparameters in the covariance of the Gaussian process to adapt the surrogate model to fine structures on top of broader extrema of the complex model. For reasons of comparison the result of an iteration solely applying the variance utility $U_{\mathrm{var}}$ is depicted for iteration step 20 in Figure 2f. Due to the local traps the optimization routine found in seven iteration steps positions for which the model data value was already obtained ($N_{\mathrm{double}}$ = 7). Actually, it takes *N* = 58 iterations until the global optimum is found this way.

An overview of how many iterations it takes to reveal the global optimum is given by Table 1.

**Figure 3:**
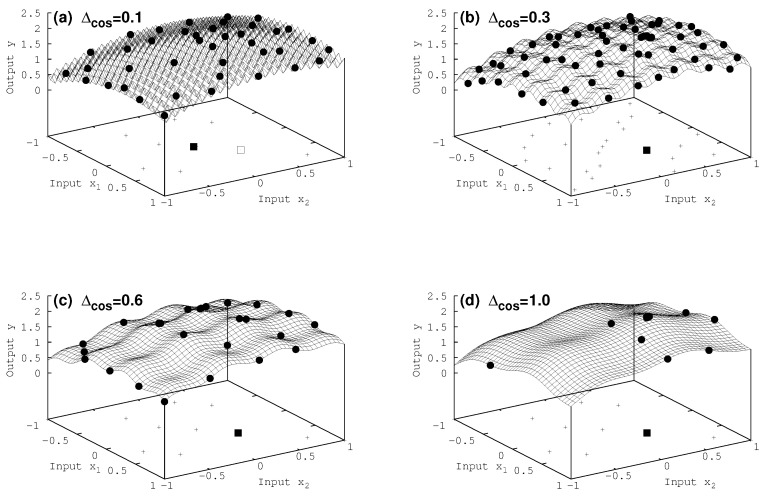
Two-dimensional case: surface of the complex model for various cosine fine structures $\Delta_{\cos}\in \left\{(a)\;0.1,(b)\;0.3,(c)\;0.6,(d)\;1.0\right\}$ The initial 10 input data are shown in the basement (plus signs), and on top of the surface the additional data points which were acquired during the iteration of the procedure (filled circles). All maxima found (filled square) are the true ones, apart from the case of $\Delta_{\cos}$ = 0.1 in (**a**), where the true maximum is represented by the open square.

In all one-dimensional cases our proposed algorithm was capable of finding it, sometimes with far fewer iterations than in the runs with applying one of the utilities. Only for $\Delta_{cos}=1.0$ the results of EI and MV are competitive to employing the combined utilities. Here the complex model is reduced to only one pronounced structure, which can be seen in Figure 3d for two dimensions (but is the same for one dimension), so no traps exist for which a sophisticated algorithm is needed to escape from. In such a case, the algorithm employing uncombined utilities performs a little bit better as with combined ones, because it is easier for the algorithm to head strongly to the extremum, without taking care of the result of the second utility. The runs were stopped as soon as 100 sequential iterations did not result in new suggested data points. The position of the largest maximum found at this time is given by the coordinates stated in the table. Of course, increasing the number of iterations without such limitation would reveal the global optimum in the end—however, at immense cost. We have to keep in mind that we need a method which autonomously finds the global optimum of an complex model expensive for our evaluations. Therefore, we want calls to the complex model to be as seldom as possible.

For the sake of comparison the results of the global optimization by a simple simulated annealing approach are shown in Table 1. Depending on the choice of the input parameters (initial temperature level, number of function evaluations per step) to the particular annealing algorithm (here amebsa, chapter 10.9 of [28]) all get detected in the one-dimensional case. Playing around, the initial temperature level had to be set somewhere between *T* = 1000 and 3000, while the number of function evaluations at each iteratively diminished temperature level had to be in between 10 and 40. However, exactly those adjustments by hand are what we want to avoid when we come to complex models with hidden extremal structures. We simply will not know when to stop the adjustments. Moreover, even worse, it takes at least a factor of ten more function calls to the complex model than our proposed algorithm needs. Though the running time with respect to the mere simulated annealing part is much less compared with our algorithm, it is this extensive calling of the complex model function which dominates the overall computing effort. Regarding our algorithm the use of a particular utility function (at least for the ones employed in this paper) is of no consequence for the running time, since the calculation of the hyperparameters with MCMC is by far the largest time-consuming part-again apart from calling the complex model function.

### 7.2. Two-Dimensional Results

Eventually, we turn in Figure 3 to the two-dimensional case. Again the true maximum of the complicated function is found, apart from $\Delta_{\cos}$ = 0.1 in Figure 3a, where the algorithm gets erroneously stuck in a local maximum close to the true one. In the later case the simulated annealing approach fails as well. It ends up in a local maximum, as can be seen in Table 1. Moreover, making use of only one of the utilities (expected improvement or maximum variane, in Table 1) seems to be even less successful. Since with smaller $\Delta_{\cos}$ the extensions of the local extrema shrink in size compared to the broader maximum in Equation (18), it gets more and more challenging to converge to the correct results. It is matter of ongoing work to characterize the interplay of local and broader extremal structures and its impact on the result with respect to the choice of the utility function.

**Figure 4:**
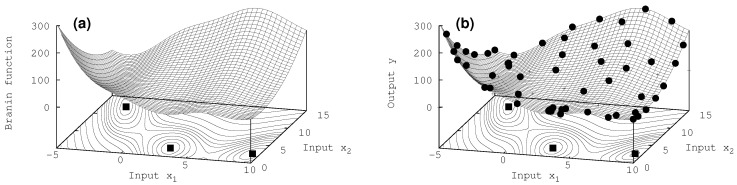
Two-dimensional case: (**a**) Branin function, (**b**) surrogate for *N* = 60 new acquired data values. The contours shown in the basement have non equidistant structure (higher resolution close to the minima). The three squares represent the exact position of the three minima. An uncertainty of $\sigma_{d}$ = 0.001 has been assigned to the data.

Finally, for the two-dimensional Branin function [30] the results are shown in Figure 4.

All three characterizing extrema at $x\in \left\{(-\pi ,12.275),(\pi ,2.275),(3\pi ,2.475)\right\}$ are revealed successfully with about 60 iterations. For such a function which prominent structure only in these three extrema, the algorithm even succeeds in getting a good overall representation of the true model by the surrogate, as can be seen by comparing the surfaces the and the contour lines in the basement of the exact Branin function Figure 4a and the surrogate model in Figure 4b.

## 8. Summary and Conclusions

An algorithm for global optimization was demonstrated to autonomously reveal dominant maxima. The Bayesian approach benefits from the self-adjusting skills of hyperparameters in the Gaussian process, which enables the surrogate model to adapt to fine structures on top of broader extrema of the complex model. To be instructive the procedure was characterized for two low-dimensional examples. The proposed method does not explore full hyperparameter space during the maximum search in the surrogate. As was shown by Osborne et al. [24], the spread of the probability distribution over the phase space of the hyperparameters may be such that the statement of an expectation value (and its variance) is not a good representation for the distribution. However, we claim that it is not necessary to use the full distribution to reveal the global optimum in an iterative process. The “iterative power” will force the convergence to the extremal structure and the number of points acquired in the vicinity of the maximum will locally pin the surrogate to be an exact representation of the true model. As a consequence, we make use of the expectation values of the hyperparameters only and profit from a quickly accessible surrogate. Contrary to other procedures [23,24] a marginalization over hyperparameters is not performed, which speeds up the search for an extremum. Especially in higher dimensions it will turn out to be of significant importance to quickly get a good idea of where to look for a maximum. This is solved in the paper by proposing the maximal value of the respective utility function from a manageable set of all points being in the middle between all possible pairs of points in the data set and additionally at all data points themselves. Then the comparably simple Powell’s method returned the position of the nearest (local) maxima to give the input parameter for the next simulation of the costly model. For all this, alternating utility functions were employed instead of a single measure as an utility function (like in [2,24]). To summarize: in order to keep the numerics quick, the algorithm makes compromises on the way but is exact on the finishing line.

It is left to ongoing research to show the feasibility of the proposed method for identifying extrema in more than two dimensions. For instance, instead of simply taking the middle inbetween neighboring data points, one has to think of more sophisticated strategies like Voronoi diagrams in order to identify initial values for the maximum search with the boundary condition of largest distance to the acquired data (within the RoI). Moreover, in the search for the maximum within the surrogate one could profit from the gradients intrinsically at hand for Gaussian processes. Even the distribution function of the gradients is available. Furthermore, one may think of other utility functions additionally employed or modifications of the used ones from Equations (15) and (16). For instance, to set a bias to extremal structures one could look at the sum of the variance Equation (9) and the expectation value Equation (8) of the surrogate instead at the variance alone. Another issue is to cope with data which are not normally distributed but have an uncertainty with student-t behaviour. There exist some rare but promising approaches in the literature [31,32] which we intent to employ. Eventually the parallel capabilities of modern computer architecture could be exploited in order to search not one but multiple surrogates for their respective maximum and select from among these which parameter set should be chosen for the most expensive generation of a new data point.

## Figures and Tables

**Table 1:** The table shows the number *N* of iterations required to find the global optimum of the Rastrigin-like true model Equation (18) for various $\Delta_{\cos}$, when employing either the expected improvement (EI) utility Equation (15), the maximum variance (MV) utility Equation (16), or their combination (EI + MV). If the global optimum could not be discovered by applying the respective utility function the position of the local maximum found is stated instead. The true model has its one-dimensional global optimum with *y* = 2.1 at *x* = 0.3, and its two-dimensional one with *y* = 2.2 at $x_{1}$ = 0.3, $x_{2}$ = 0.3. Results of a standard simulated annealing method (see [28]) are provided in order to compare the expenditure of calls to the complex model function. See Preuss et al. [29] for additional figures depicting the results for $\Delta_{\cos}=0.1$. and $\Delta_{\cos}=1.0$.

Utility	$\Delta_{\cos}$ = 0.1	$\Delta_{\cos}$ = 0.3	$\Delta_{\cos}$ = 0.6	$\Delta_{\cos}$ = 1.0
1D, $\sigma_{d}$ = 0.01				
EI	*x* = 0.54, *y* = 2.02	*x* = 0.59, *y* = 2.06	*N* = 17	*N* = 15
MV	*x* = 0.07, *y* = 2.01	*N* = 58	*N* = 21	*N* = 13
EI + MV	*N* = 112	*N* = 20	*N* = 10	*N* = 15
1D, $\sigma_{d}$ = 0.001				
EI	*x* = −0.01, *y* = 2.06	*x* = 0.68, *y* = 2.06	*x* = 0.25, *y* = 2.09	*N* = 6
MV	*x* = 0.47, *y* = 1.99	*N* = 22	*N* = 11	*N* = 8
EI + MV	*N* = 9	*N* = 34	*N* = 10	*N* = 8
Annealing	*N* ≈ 700	*N* ≈ 1100	*N* ≈ 700	*N* ≈ 600
2D, $\sigma_{d}$ = 0.001				
EI	x = (0.1, 0.1), *y* = 2.1	x = (0.0, 0.0), *y* = 2.1	x = (−0.2, −0.2), *y* = 1.9	x = (0.3, 0.3), *y* = 2.2
MV	x = (−0.1, 0.1), *y* = 2.2	x = (0.4, 0.1), *y* = 2.2	*N* = 84	*N* = 70
EI + MV	x = (0.0, 0.0), *y* = 2.2	*N* = 105	*N* = 50	*N* = 23
Annealing	x = (0.4, 0.5), *y* = 2.2	*N* ≈ 1200	*N* ≈ 1100	*N* ≈ 800

## Appendix A. Notation Table

*N*	number of input data vectors
$N_{\dim}$	number of elements in the input data vector
$N_{\mathrm{data}}$	number of points added to the data pool by optimization
$N_{\mathrm{double}}$	number of points for which standard error was reduced
$x_{*}$	test input vector
$x_{i}=\left(x_{i1},\ldots ,x_{iN_{\dim}}\right)$	*i*-th input data vector
$X=(x_{1},x_{2},\ldots ,x_{N})$	$N\times N_{dim}$ matrix with input data vectors as columns
$X^{\prime }=\left\{X,\xi \right\}$	matrix of the input data vectors expanded by the vector of grid points
ξ	vector of grid points within region of interest I
$\xi_{\max}$	grid point with largest utility
$f_{*}$	target value at test input vector $x_{*}$
f(x)	function of input data to describe target data
$f_{\max}$	maximum of the surrogate model function
$y={\left(y_{1},\ldots ,y_{N}\right)}^{T}$	vector of the *N* target data
ϵ	uncertainty of the target data
${\sigma_{d}}_{i}^{2}$	variance of the *i*-th target data
$\Delta_{ij}={\sigma_{d}}_{i}^{2}\delta_{ij}$	ij-th element of the N×N matrix of the variances of target data
λ	length scale to set up the notion of distance between input data vectors
$\sigma_{f}^{2}$	signal variance of the distribution over functions *f*
$\sigma_{n}^{2}$	overall noise in the data
$\theta =(\lambda ,\sigma_{f},\sigma_{n})$	vector of the hyperparameters
$k(x_{p},x_{q})$	covariance of two input data vectors
${\left(k_{*}\right)}_{i}=k\left(x_{*},x_{i}\right)$	short notation for the *i*-th element of the vector of covariances between test
	input vector and input data vector
$K_{pq}=k\left(x_{p},x_{q}\right)$	pq-th element of the N×N covariance matrix of the input data vectors
$K^{\prime }$	(N+1)×(N+1) covariance matrix of the expanded input $X^{\prime }$
RoI	region of interest to run Gaussian processes
*I*	improvement of the maximum $f_{\max}$ of the surrogate model
U(ξ)	utility of a target data obtained at input vector ξ
